# Physicochemical composition, lipid oxidation, and microbiological quality of ram mortadella supplemented with *Smallanthus sonchifolius* meal

**DOI:** 10.1002/fsn3.1880

**Published:** 2020-09-17

**Authors:** Alexandre Cristiano do Santos Junior, Rodrigo Fortunato de Oliveira, Fabio da Costa Henry, Jonhny de Azevedo Maia Junior, Monique Moreira Moulin, Suzana Maria Della Lucia, Célia Raquel Quirino, Meire Lelis Leal Martins, Maria Cecília Cabral Rampe

**Affiliations:** ^1^ Federal Institute of Espírito Santo Alegre Brazil; ^2^ North Fluminense State University Darcy Ribeiro Campos dos Goytacazes Brazil; ^3^ Departamento de Engenharia de Alimentos Universidade Federal do Espírito Santo (UFES) Alegre Brasil

**Keywords:** food safety, fructooligosaccharides, lamb, meat products

## Abstract

This study evaluated the physicochemical, lipid peroxidation, and microbiological quality of mortadellas prepared with ram and supplemented with different amounts of *Smallanthus sonchifolius* meal. Three mutton mortadella formulations supplemented with 1.25%, 2.50%, and 5% yacón meal and control formulation without yacón meal was included. The physicochemical, lipid peroxidation, and microbiological analyses were carried out in the time periods 10, 45 days, three, and six month after the preparation of mortadella. The control formulation presenting lighter and more intense red tone compared with the other formulations. All formulations presenting lipid peroxidation increased 90 days after processing; already the pH and Aw values were constant for all formulations at the experimental times stipulated. All formulations had the physicochemical characterization and microbiological quality standards, according to defined in regulations for mortadella production in Brazil. The results show that mutton mortadella supplemented with yacón meal is a promising alternative in the manufacture of healthy meat products.

## Graphical abstract

Mutton mortadella supplemented with yacón meal is a promising alternative in the manufacture of healthy meat products. Compounds present in yacón meal also play a role in the color of mortadella formulations and are used together with antioxidants, improving flavor, maintaining sensory quality, and prolonging shelf life.

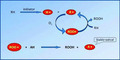

## 1 INTRODUCTION

Aiming to meet the demands and preferences of increasingly health‐conscious customers, a considerable number of studies currently addresses the development of more healthy foods (Biswas, Kumar, Bhosle, Sahoo, & Chatli, 2011; Vidal et al., 2012; Cardoso, Henry, Almeida, Ferreira, & Ladeira, 2013; Doménech‐Asensi et al., 2013; Maia Junior et al., 2013). In this scenario, much research has been dedicated to conceive innovative formulations of meat products. More specifically, such innovation efforts are directed toward the development of products that may be considered functional, that is, foods that exert beneficial health effects in addition to nutrition potential, becoming useful alternatives in the prevention and treatment of diseases (Dutra et al., 2013; Leite et al., 2015; Méndez‐Zamora et al., 2015; Viuda‐Martos, Ruiz‐Navajas, Fernández‐López, & Pérez‐Álvarez, 2010; Yetim, Gokalp, Kaya, Yanar, & Ockerman, 1992).

Mortadella is one of the most consumed cooked sausages by the world population, being processed using mainly pork, but it can be made from meat of various species. Today mutton emerges as a feasible alternative on development from coated with skin stuff owing to the great nutritional and sensory quality besides the fact that it is not the object of religious restrictions. Mutton has high levels of protein and ironwork and suitably lower levels of fat, compared withal other meat kinds, gaining preference as ingredient of healthier skin‐encased products (Lima Júnior, Rangel, Urbano, & Moreno, 2013; Osório, Osório, & Sañudo, 2009). Several studies are investigating the incorporation of sheep or goat meat in sausages and the use of natural antioxidants as protective factors against lipid oxidation. A study was conducted using different levels of pork fat in goat mortadella (Guerra et al., 2011), using jabuticaba peel extract at 0.5%, 0.75%, and 1.0% showed high antioxidant power (Almeida et al., 2015), using tomato paste increased product stability and shelf life by significantly reducing lipid oxidation associated with storage (Doménech‐Asensi et al., 2013), and using fruit aqueous extract of the Brazilian pepper tree in fresh pork sausage that best resisted lipid peroxidation (Oliveira et al., 2020).

The main strategy used by the meat industry to inhibit lipid oxidation is the addition of antioxidants to meat and meat products (Cunha et al., 2018; Echegaray et al., 2018; Gómez et al., 2018; Lorenzo et al., 2018; Pateiro et al., 2018; Zamuz et al., 2018). However, nowadays consumers demand more natural products, which limits the industry in their use of currently allowed synthetic antioxidants in foods, leaving manufacturers with few options (Barden & Decker, 2016). As a source of bioactive composts like fructooligosaccharides, inulin, and phenolic composts, the flour obtained from the tuberous root yacón (*Smallanthus sonchifolia*) stands as an alternative on fabrication to healthfull skin‐encased products and is now considered a functional food (Delgado, Tashiro, Maróstica Júnior, & Pastore, 2013; Ojansivu, Ferreira, & Salminen, 2011). The explanation is that the human body makes not yield enzymes which metabolize fructooligosaccharides, which therefore goes through the stomachic track unmetabolized, providing small energy levels and becoming a promising ingredient in foods to obese individuals with associated transmissible diseases (Borges et al., 2012). In addition, it should be highlighted that yacón meal is produced by dehydration, which makes it even more interesting as a food ingredient due to the resulting increased shelf life and the possibility to add it as supplement to a variety of other foods.

Like any other food, the quality of meat products is assessed based on sensory, physicochemical, and microbiological attributes. Yet, percent composition, instrumental analyses that include pH, water activity (Aw), color, and texture, as well as the determination of lipid oxidation level also are important parameters supplying essential information concerning nutritional quality and as well checking the shelf life of the product (Gomes & Oliveira, 2011). For example, as one of the chemical reactions most often observed in foods, lipid peroxidation is the main cause behind the deterioration of several food products. These reactions are triggered by contact with oxygen, when electrons are transferred between molecules and free radicals are produced, oxidizing lipids. The list of negative outcomes of lipid peroxidation includes undesired changes in flavor, smell, overall appearance, physical attributes, and nutritional value, besides shorter shelf life. Levels of thiobarbituric acid (TBARS) are the main parameter to measure lipid peroxidation in foods (Araújo, 2011).

The control of microbiological contamination is key to guaranteeing consumer safety and establishing a food product's shelf life. Importantly, like any other food product, mortadella is subject to the action of harmful microorganisms, some of which are pathogenic, which reduces shelf life and increases the risk of food‐borne diseases (Franco & Landgraf, 2005). In addition, there is a nutritional loss that leads to the formation of toxic substances, so the control of oxidative processes is of vital importance for the meat industry (Domínguez et al., 2019).

On these bases, study proposes to evaluate the possible effects of storage on the quality of mortadella formulations made with ram and supplemented with different amounts of *Smallanthus sonchifolius* meal at several stages of the production process.

## 2 MATERIALS AND METHODS

### 2.1 Raw materials

The mutton shoulder cuts used were from Santa Inês and Dorper sheep bred in confinement. All animals were slaughtered in a commercial slaughterhouse (Atílio Vivácquua, ES, Brazil) which is inspected the by the State Inspection Service.

Mutton cuts were stocked in a freezer at −18ºC upon the production in mortadellas. *Smallanthus sonchifolius* meal and the some other ingredients used in formulations were purchased on specialized stores selling native crop products and chemical additives for skin‐encased products.

### 2.2 Mortadellas of ram

Four experimental mortadella formulations (F1, F2, F3, Control) were prepared with mutton and different amounts of yacón meal. Four replicates were performed for each formulation.

Preliminary assays and a review of current regulations regarding food quality and identity standards were conducted to define the percent composition of raw materials and other additives in addition to fat reduction percent values and the respective amounts of yacón meal supplemented to mortadella formulations (Allais, 2010; Brasil, 2000; Guerra et al., 2012; Yetim et al., 1992). Percent composition of formulations is presented as percent raw material and additives values on Table 1.

**TABLE 1:** Formulations used in the elaboration of mutton mortadella supplemented with *Smallanthus sonchifolius* meal

Raw material	Control	F1	F2	F3
Mutton	67.00	67.00	67.00	67.00
Fat	30.00	27.00	24.00	21.00
Yacón meal^a^	0.00	1.25	2.50	5.00
Sugar^b^	0.10	0.10	0.10	0.10
Salt (NaCl)^c^	2.10	2.10	2.10	2.10
Sodium nitrite^d^	0.20	0.20	0.20	0.20
Emulsifier^e^	0.30	0.30	0.30	0.30
Color fixer^f^	0.30	0.30	0.30	0.30
Water	0.00	1.75	3.50	4.00

^a^ Natural Life^®^ yacón meal.

^b^ União^®^ sugar.

^c^ Cisne^®^ salt.

^d^ Kura K007 ‐ Doremus^®^ curing salt.

^e^ Ibrac^®^ emulsifier.

^f^ Ibrac^®^ color fixer.

Mortadella formulations were processed according as Guerra et al. (2011) withal alterations. Initially, the raw material was allowed to thaw in a BOD incubator (Eletrolab EL101/3 250W, Eletrolab, São Paulo, SP, Brazil) at 4 ± 0.5ºC. After thawing, ram and pork grease were ground using a beef griding machine (Caf 8 Inox) withal grind plates with 8‐mm, 5‐mm, and 3‐mm disks. Immediately after mutton, pork grease, Smallanthus sonchifolius meal, and the some other additives were weighted and mixed on a multiprocessor (Philips RI7636 750W) for approximately 6 min upon complete homogenization of the material. After processing, the formulations were separated and packed in polyethylene bags to cook mortadella.

The samples were cooked on an stainless steel former on an dual bathe at 80ºC to the temperature in the middle of mass achieve 74ºC. Mortadellas were separated, tagged, vacuum‐packed (Selovac 200‐B, São Paulo, SP, Brazil) in polyethylene bags at 4 ± 0.5ºC until analysis and shelf‐life estimation during 120 days after processing.

### 2.3 Physicochemical characterization

Four samples from each one formulation were ground, combined, and used in the following analyses: (a) moisture (difference between the weights of the samples before and after drying in a stove at 105ºC), (b) mineral matter, (c) protein concentration (classic Kjeldahl method), (d) fat content (direct Soxhlet method), (e) dietary fiber (acid digestion and gravimetric analysis), and (f) non‐nitrogen content (by subtraction). All analyses were carried as recommended by to Cechi (1999) and AOAC (2000).

### 2.4 Water activity, pH, and mortadella color

Mortadella subsamples were inserted into the container (with a capacity of 15 ml) of Aqualab (Decal Devices Inc.) water activity analyzer (Aw), which uses the dew point principle. In the pH analyses, a 10‐g sample of mortadella was first homogenized in 100 ml distilled water with shaking for 5 min, and pH was determined in a potentiometer (Schott Handylab).

Color (lightness—L*; redness—a*; and yellowness—b*) was determined immediately after the samples were processed, using a colorimeter (Minolta CR‐400; Konica Minolta Sensing, Inc., Osaka, Japan) (settings: diffuse illumination/0º viewing angle, illuminant D65, specular component included) calibrated to a white standard. The raw samples were cut in half and the equipment was placed at three different points in the mortadella mass. The saturation index was calculated using the equation C* = (a*2 + b*2)1/2.

### 2.5 Lipid peroxidation

TBARS levels indicate lipid peroxidation. Thiobarbituric acid is used to quantify levels of malondialdehyde (MDA), a short‐chain aldehyde formed by the decomposition of lipid hydroperoxides produced by oxidation reactions. The analyses of mortadella formulations were carried out according to the methodology described by Vyncke (1970) and modified by Sorensen and Jorgensen (1996). Readings were conducted in a spectrophotometer (UV‐5100, Kasuaki) at 532 nm against a concentration (x):absorbance (y) curve of 1,1,3,3‐tetraethoxypropane, used as standard. Values were expressed as mg MDA/kg.

### 2.6 Microbiological analyses

Microbiological analysis of the samples was performed, according to Silva Junqueira & Silveira (2007). The results were compared with the standards recommended in the RDC 12 (Brasil, 2001), for coliforms at 45ºC (Most Probable Number method), Coagulase‐positive Staphylococcus (Presence or absence), Salmonella spp. (presence or absence) and sulfite‐reducing clostridia at 46ºC.

### 2.7 Statistical analyses

The data obtained in the physicochemical analyses were analyzed as a completely randomized experimental design with four treatments and three repeats each. The results were submitted to analysis of variance (ANOVA) at 5% probability. The treatments were compared by the Student–Newman–Keuls test (SAS, 2009 version 9.2).

## 3 RESULTS AND DISCUSSION

Mean chemical composition of mortadellas (humidity, total protein, lipid, ash, dietary fiber, and non‐nitrogen content) of ram mortadella formulations are presented in Table 2.

**TABLE 2:** Physicochemical parameters and yield (mean ± standard deviation) of mortadella formulations prepared with mutton and supplemented with different amounts of *Smallanthus sonchifolius* meal

Parameters	Formulations			
	Control	F1	F2	F3
Moisture (%)	56.40 ± 0.10^a^	55.00 ± 0.10^b^	55.16 ± 0.15^b^	54.60 ± 0.15^c^
Proteins (%)	16.00 ± 0.00^c^	18.23 ± 0.06^b^	18.43 ± 0.40^ba^	18.80 ± 0.10^a^
Fat (%)	25.10 ± 0.10^a^	23.73 ± 0.15^b^	22.03 ± 0.38^c^	20.13 ± 0.15^d^
Ash (%)	2.50 ± 0.00^b^	2.50 ± 0.00^b^	2.60 ± 0.00^b^	3.10 ± 0.10^a^
Dietary fiber (%)	0.00 ± 0.00^d^	0.30 ± 0.00^c^	0.40 ± 0.00^b^	1.13 ± 0.06^a^
Non‐nitrogenated content (%)	0.00 ± 0.00^d^	0.24 ± 0.06^c^	1.38 ± 0.06^b^	2.24 ± 0.06^a^
Yield (%)	67.83 ± 1.05^b^	68.30 ± 1.20^b^	72.06 ± 3.05^a^	73.70 ± 1.10^a^

* Values followed by different lowercase letters on the same line indicate statistically significant differences (*p* < .05) in the Student–Newman–Keuls test.

Moisture values varied between 54.60% and 56.40%, proteins from 16.00% to 18.80%, fat between 20.13% and 25.10%, ash between 2.50% and 3.10%, fiber from zero to 1.13%, and non‐nitrogenated contents ranged from zero to 2.24%. The results show that the use of fat in formulations affected moisture, protein, and fat content values significantly (*p* < .05) between mortadella formulations.

On the whole, moisture values decreased with higher yacón meal percent values and lower fat levels in formulations. Leite et al. (2015) also observed that frankfurters with high moisture values were prepared with the lowest fat contents. Regarding the moisture content, it is worth noting that the use of potato yacon flour was proportionally higher in the formulations F1, F2, and F3, and the proportional addition of water in these formulations was necessary in order to avoid changes in the texture of the samples due to the high capacity of water retention (WHC) of yacon potato flour. Moisture contents in all samples were beneath the 65% maximum admissible level established in Brazilian regulations.

Despite the statistically significant differences in protein levels between formulations (*p* < .05), every met the minim 12% value stated in Brazilian legislation. How stabilizing agent in the elaboration of emulsified products, proteins, are active on the lipid–water interface, cutting interface pressure, and enriching mixing to ingredients. Proteins also prevent the coalescence and loss of lipids, playing an essential role concerning acceptable sensory traits of emulsified skin‐encased products (Guerra et al., 2012).

Fat levels also differed significantly between formulations (*p* < .05). Although this result was expected due to the decreasing amounts used in F1, F2, and F3, in that order, all formulations met the 30% maximum admissible level defined on Brazilian norms (Brasil, 2000). For Guerra et al. (2012), fat levels are extremely important to ensure that the product will have its peculiar sensory traits, directly affecting texture and taste.

The results of the study also show as increasing amounts of yacón meal induced significant differences (*p* < .05) in ash, dietary fiber, and non‐nitrogenated contents, improving yield of all formulations, compared withal the control, that did not added *Smallanthus sonchifolius* meal. According to Mendoza, García, Casas, and Selgas (2001) and Barreto Pacheco & Pollonio (2015), the addition of dietary fiber to meat products may improve yield, reducing costs of a formulation, improving texture, and influencing rheological and sensory behavior, especially appearance and color.

Overall, the percent composition of the formulations developed in the present study is similar in the data acquired in various cooked skin‐encased products prepared withal mutton and *Smallanthus sonchifolius* meal and flour of some other vegetables (Huang, Tsai, & Chen, 2011; Dutra et al., 2013; Barreto, Pacheco, & Pollonio, 2015; Contado, Rocha, Queiroz, Abreu, & Ramos, 2015; Santos Júnior et al., 2017).

The variation of pH of mortadella formulations prepared with mutton and *Smallanthus sonchifolius* meal 120 days behind processing varied between 5.86 and 6.11. Overall, all formulations exhibited similar pH curves. Interestingly, Aw values did not vary statistically between formulations throughout the experiment, remaining constant at 0.97 (*p* > .05). For Guerra et al. (2011), pH and Aw are important quantitative parameters in cooked skin‐encased meat products, since high values promote the development of undesired microorganisms, while low figures affect texture and flavor negatively.

The color curves of formulations constructed with data obtained on days 10, 45, 90, and 120 days after processing are shown in Table 3.

**TABLE 3:** Color (means ± standard deviation) of mortadella formulations prepared with mutton and supplemented with different amounts of *Smallanthus sonchifolius* meal after different storage periods

Parameters/days	Formulations			
	Control	F1	F2	F3
L*				
10 days	59.84 ± 0.34^Aa^	56.02 ± 1.60^Ab^	54.34 ± 0.69^Ac^	51.03 ± 0.20^Ad^
45 days	60.18 ± 0.67^Aa^	56.86 ± 0.19^Ab^	55.14 ± 0.47^Ac^	51.41 ± 0.32^Ad^
90 days	58.35 ± 0.16^Ba^	54.10 ± 1.51^Bb^	51.73 ± 0.63^Bc^	50.30 ± 0.31^Bd^
120 days	55.57 ± 1.48^Ca^	55.68 ± 1.68^Ba^	52.38 ± 0.80^Bb^	50.12 ± 0.21^Bc^
a*				
10 days	12.13 ± 0.04^Aa^	11.42 ± 0.20^Ab^	10.59 ± 0.03^Ac^	9.97 ± 0.11^Ad^
45 days	12.09 ± 0.21^Aa^	10.84 ± 0.13^Bb^	10.13 ± 0.18^Bc^	8.26 ± 0.15^Bd^
90 days	11.90 ± 0.12^Aa^	10.95 ± 0.06^Bb^	10.37 ± 0.12^Bc^	7.89 ± 0.09^Cd^
120 days	11.97 ± 0.21^Aa^	9.79 ± 0.15^Cb^	8.63 ± 0.06^Cc^	6.56 ± 0.38^Dd^
b*				
10 days	12.37 ± 0.37^Ad^	13.35 ± 0.18^Ac^	14.27 ± 0.31^Ab^	16.11 ± 0.27^Aa^
45 days	12.10 ± 0.30^Ac^	13.50 ± 0.34^Ab^	14.01 ± 0.12^Aa^	14.07 ± 0.17^Ba^
90 days	12.24 ± 0.17^Ac^	13.07 ± 0.15^Ab^	14.20 ± 0.31^Aa^	12.75 ± 1.28^Cb^
120 days	12.04 ± 0.44^Ab^	12.92 ± 0.34^Aa^	12.63 ± 0.15^Ba^	11.89 ± 0.82^Cc^
c*				
10 days	17.41 ± 0.30^Ab^	17.54 ± 0.24^Ab^	17.81 ± 0.28^Ab^	18.94 ± 0.29^Aa^
45 days	17.10 ± 0.35^Aa^	17.39 ± 0.23^Aa^	17.58 ± 0.27^Aa^	16.33 ± 0.21^Bb^
90 days	16.94 ± 0.42^Ab^	16.99 ± 0.05^Bb^	17.29 ± 0.14^Aa^	15.00 ± 0.41^Cc^
120 days	16.19 ± 0.20^Ba^	16.21 ± 0.35^Ca^	15.30 ± 0.08^Bb^	13.59 ± 0.42^Dc^

* Values followed by different capital letters in the same column and values in lowercase letters on the same line indicate statistically significant differences (*p* < .05) in the Student–Newman–Keuls test.

In the color analysis, L* values (which represent luminosity; 0 = black, 100 = white) of formulations differed statistically (*p* < .05). The results show that the highest the quantity of *Smallanthus sonchifolius* meal admixed, the murker were the mortadella formulations. While F3, which was prepared with the highest amount of yacón meal exhibited the murkest color, the control formulation (with no yacón meal) presented the lightest. It was also observed that all formulations became mildly darker as experimental time advanced, with statistically significant differences (*p* < .05) 90 days after processing.

Also, the admixture of different fat levels also affected color, making lighter mortadella, which may therefore explain the differences in L* values observed for formulations. Sánches‐Rodríguez (2001) claimed that water and lipid contents may affect L* values considerably, as observed in the present study. For example, the control formulation, which contained no yacón meal and had the highest amount of added fat, presented the lightest hue.

Indicating intensity of red hues, a* values differed between samples throughout the experiment (*p* < .05). This difference was directly proportional to the amount of yacón meal added to each formulation, when high yacón meal contents produced lighter mortadella. Moreover, the redness decreased in all experimental formulations with time and differed statistically from the control formulation (*p* < .05).

Statistically significant difference (*p* < .05) was also observed for b* values between formulations during the experiment. The highest the quantity of *Smallanthus sonchifolius* meal added, the highest the b* values of formulations were. But a decrease in b* values was also observed, only for F2 and F3 on days 120 and 45 after processing. It should be highlighted that b* values express intensity of yellow color. Yet, in the analysis of meat products the parameter represents brown hues (Barreto, Pacheco, & Pollonio, 2015). Thus, F2 and F3, which were made with the intermediate and the high contents of *Smallanthus sonchifolius* meal, respectively, presented rather intense brown hues. The effect of redness and yellowness gets rather apparent when the parameters are measured based on the saturation index, C*. Overall, C* values decreased (*p* < .05) for all formulations during 120 days into storage.

For Zeola Souza Souza Silva Sobrinho & Barbosa (2007), color of skin‐encased products is the most important quality parameter the consumer assesses as of purchase, determining the choice made. Overall, the control formulation did was the light and reddest, while the other formulations became darker and lost intensity of red color with time, that can affect sensory acceptability. However, the overall color effects acquired were like, as observed by Contado Rocha Queiroz Abreu & Ramos (2015) in a investigation which tested ham‐like meat products formulated with *Smallanthus sonchifolius* meal.

The TBARS values of the mortadella formulations prepared and analyzed in the present study are shown in Table 4.

**TABLE 4:** TBARS of mortadella formulations prepared with mutton and supplemented with different amounts of *Smallanthus sonchifolius* meal after different storage periods

Parameters/days	Formulations			
	Control	F1	F2	F3
10 days	0.007 ± 0.00^Dc^	0.011 ± 0.00^Db^	0.012 ± 0.00^Bb^	0.013 ± 0.00^Ba^
45 days	0.010 ± 0.00^Cd^	0.019 ± 0.00^Cc^	0.021 ± 0.00^Ab^	0.028 ± 0.00^Aa^
90 days	0.031 ± 0.00^Ba^	0.031 ± 0.00^Ba^	0.021 ± 0.00^Ac^	0.027 ± 0.00^Ab^
120 days	0.070 ± 0.00^Aa^	0.043 ± 0.00^Ab^	0.022 ± 0.00^Ad^	0.027 ± 0.00^Ac^

* Values followed by different capital letters in the same column and values in lowercase letters on the same line indicate statistically significant differences (*p* < .05) in the Student–Newman–Keuls test.

The parameter is the most commonly used to determine lipid peroxidation in meat products, when MDA levels in samples reach between 0.5 mg/kg and 2.0 mg/kg. MDA levels in mortadella formulations remained constant until day 10 after processing, with no statistically significant differences (*p* > .05). Starting on day 90 into storage, TBARS levels increased in all formulations. But on day 120, TBARS levels of F1 and the control formulation were significantly higher (*p* < .05) compared with F2 and F3, which were prepared with the intermediate and high levels of yacón meal used in this study.

Despite the variation in lipid peroxidation levels, the TBARS values obtained indicate that the addition of yacón meal did not significantly influence the fast oxidative changes observed in products soon after processing. But 120 days after processing, F2 and F3 had lower TBARS values, showing that yacón meal may in fact be a useful ingredient to prevent oxidative changes during the storage of mortadella.

Lipid peroxidation is an inevitable chemical phenomenon observed during the storage of meat products. However, it may be inhibited adding antioxidants that lend greater stability, preventing the oxidative rancidness of fats and changes in sensory attributes. In this sense, yacón meal is an interesting source of phenolic compounds, mainly chlorogenic acid, which may play an antioxidant role (Santana & Cardoso, 2008). Furthermore, the antioxidant power of phenolic compounds has been associated with the hydroxyl group bound to the aromatic ring, to which it donates the electrons of its hydrogen atom, neutralizing free radicals (Radha Krishnan et al., 2014).

The results of the present study are similar to those published by Teixeira (2010) that confirmed the antioxidant effect of yacón meal in ham‐like skin‐encased products. For Wu Duckett Neel Fontenot & Clapham (2008) and Prior et al. (2003), this antioxidant effect of yacón meal is induced by heating or cooking mortadella formulations, which in turn promotes proteolysis. In addition to that, the actual proteins present in mortadella formulations may have antioxidant effect as well. For example, the dipeptide carnosine has a hydrophilic group whose antioxidant activity is comparable to that of ferulic acid, a phenolic compound. It may therefore be assumed that the proteolysis reactions occurring during storage are behind the similar antioxidant activity of samples of mortadella formulations in the present study. The exceptions were F1 and the control formulation, which had higher antioxidant activity values on day 120 after processing. It should be emphasized that these formulations contained comparatively high levels of fat, making them more vulnerable to lipid peroxidation.

Plant‐based curing agents have been signaled as an alternative replacement of chemical additives in cured food products (Santamaria, 2006). One of the advantages of plant extracts is the ability to improve quality and shelf life of products with no need for other additives. The antioxidant effect of these compounds stabilizes fat, preventing rancidness. In addition, this effect is equivalent to that exhibited by conventional, commercial products, making them a healthier ingredient of meat products.

Mean bacterial counts of thermotolerant coliforms, coagulase‐positive Staphylococcus, sulfite‐reducing Clostridium, and Salmonella sp. are shown in Table 5.

**TABLE 5:** Microbiological analysis (means ± standard deviation) of mortadella formulations prepared with mutton and supplemented with different amounts of *Smallanthus sonchifolius* meal after different storage periods

Parameters	Time	Formulations				
		Control	F1	F2	F3	Regulations^c^
Thermotolerant coliforms^a^	10 days	<0.3	<0.3	<0.3	<0.3	<10^3^
	45 days	<0.3	<0.3	<0.3	<0.3	<10^3^
	90 days	<0.3	<0.3	<0.3	<0.3	<10^3^
	120 days	<0.3	<0.3	<0.3	<0.3	<10^3^
Coagulase‐positive *Staphylococcus* ^b^	10 days	< 1.0	<1.0	<1.0	<1.0	3 × 10^3^
	45 days	<1.0	<1.0	<1.0	<1.0	3 × 10^3^
	90 days	<1.0	<1.0	<1.0	<1.0	3 × 10^3^
	120 days	<1.0	<1.0	<1.0	<1.0	3 × 10^3^
Sulfite‐reducing *Clostridium* at 46ºC^b^	10 days	<1.0	<1.0	<1.0	<1.0	5 × 10^2^
	45 days	<1.0	<1.0	<1.0	<1.0	5 × 10^2^
	90 days	<1.0	<1.0	<1.0	<1.0	5 × 10^2^
	120 days	<1.0	<1.0	<1.0	<1.0	5 × 10^2^
*Salmonella* sp.	10 days	absent	absent	absent	absent	absence
	45 days	absent	absent	absent	absent	absence
	90 days	absent	absent	absent	absent	absence
	120 days	absent	absent	absent	absent	absence

^a^ Given as most probable number (MPN) per gram

^b^ Given as colony forming units (CFU) per gram

^c^ BRASIL. 2001.

Mortadella is a cooked meat product with a shelf life of 60 days when stocked at 4ºC. In the actual study, every microbiological count was below the maximum admissible amounts determined by Brazilian norms 120 days in storage. Such results are likely to what has been noticed in some other works (Cardoso et al., 2013; Guerra et al., 2011; Brasil, 2001).

The good microbiological quality of the mortadella formulations analyzed is an immediate result of the virtue of the raw materials used, beyond the introduction of good production practices, the utilization of additives that avoid the growing of deleterious microorganisms, and the vacuum‐packaging process employed to mortadellas.

Brazilian regulations require the use of curing salts like potassium or sodium nitrite or nitrate, for instance, in the formulations of meat products to ensure microbiological safety, reduce lipid and protein peroxidation, and improve sensory attributes. But curing agents, like compounds present in yacón meal, for instance, also play a role in the color of mortadella formulations and are used together with antioxidants improve flavor, maintaining sensory quality, and prolonging shelf life (Brasil, 2000; Pardi, Santos, Souza, & Pardi, 1996).

All formulations had the physicochemical characterization and microbiological quality standards defined in regulations for mortadella production in Brazil. The color of the control formulation was lighter and had a* tone more intense, compared with the other formulations, which became darker and less red with time. All formulations presenting lipid peroxidation increased 90 days after processing, already the pH and Aw values were constant for all formulations at the experimental times stipulated. The results show that mutton mortadella supplemented with yacón meal is a promising alternative in the manufacture of healthy meat products. It is worth noting that projects involving partnerships between research centers and the food industries become a necessity for the search for innovations, aiming to meet the demands of consumers for healthier products and make them available on a commercial scale.

## CONFLICT OF INTEREST

The authors declare that there are no conflicts of interest.

## References

[fsn31880-bib-0001] Allais, I. (2010). Emulsification In Tóldrá F. (Ed.), Handbook of meat processing (pp. 143–168). Iowa: Wiley‐Blackwell.

[fsn31880-bib-0002] Almeida, P. L. , Lima, S. N. , Costa, L. L. , Oliveira, C. C. , Damasceno, K. A. , Santos, B. A. , & Campagnol, P. C. (2015). Effect of jabuticaba peel extract on lipid oxidation, microbial stability and sensory properties of Bologna‐type sausages during refrigerated storage. Meat Science, 110, 9–14. 10.1016/j.meatsci.2015.06.012 26156583

[fsn31880-bib-0003] Araújo, J. M. A. (2011). Química de Alimentos: Teoria e Prática (5th ed). Viçosa, MG: Editora UFV.

[fsn31880-bib-0004] Association of official analytical chemists (2000). Official methods of analysis of the AOAC International. Virginia: AOAC.

[fsn31880-bib-0055] Barden, L. , & Decker, E. A. (2016). Lipid oxidation in low‐moisture food: A review. Critical Reviews in Food Science and Nutrition, 56, 2467–2482. 10.1080/10408398.2013.848833 24279497

[fsn31880-bib-0005] Barreto, A. C. S. , Pacheco, M. T. B. , & Pollonio, M. A. R. (2015). Effect of the addition of wheat fiber and partial pork back fat on the chemical composition, texture and sensory property of low‐fat bologna sausage containing inulin and oat fiber. Food Science and Technology, 35, 100–107. 10.1590/1678-457X.6496

[fsn31880-bib-0006] Biswas, A. K. , Kumar, V. , Bhosle, S. , Sahoo, J. , & Chatli, M. K. (2011). Dietary fibers as functional ingredients in meat products and their role in human health. International Journal of. Livestock Production, 2, 45–54.

[fsn31880-bib-0007] Borges, J. T. S. , Pirozi, M. R. , Paula, C. D. , Vidigal, J. G. , Silva, N. A. S. , & Caliman, F. R. B. (2012). Yacon na alimentação humana: Aspectos nutricionais, funcionais, utilização e toxicidade. Scientia Amazonia, 1, 3–16.

[fsn31880-bib-0008] Ministério da Agricultura e Abastecimento . (2000). Instrução normativa n.4, de 31 de março de 2000. Regulamentos técnicos de identidade e qualidade de carne mecanicamente separada, de mortadela, de linguiça e de salsicha. Brasilia: Ministério da Agricultura e Abastecimento. Retrieved from http://sistemasweb.agricultura.gov.br/sislegis/action/detalhaAto.do?method=consultarLegislacaoFederal

[fsn31880-bib-0009] Brasil (2001). Agência Nacional de Vigilância Sanitária, RDC nº 12, de 02 de janeiro de 2001. Regulamento técnico sobre padrões microbiológicos para alimentos. Retrieved from http://portal.anvisa.gov.br/documents/33880/2568070/RDC_12_2001.pdf/15ffddf6 3767‐4527‐bfac‐740a0400829b

[fsn31880-bib-0010] Cardoso, J. B. N. , Henry, F. C. , Almeida, S. B. , Ferreira, K. S. , & Ladeira, S. A. (2013). Characterization of cooked ham containing pectin and potassium chloride. Journal of Food Processing and Preservation, 37, 100–108. 10.1111/j.1745-4549.2011.00625.x

[fsn31880-bib-0011] Cechi, H. M. (1999). Fundamentos teóricos e práticos em análise de alimentos. Campinas, SP: Editora da Unicamp.

[fsn31880-bib-0012] Contado, E. W. N. F. , Rocha, D. A. , Queiroz, E. R. , Abreu, C. M. P. , & Ramos, E. M. (2015). Use of yacon flour and fructan extract in the formulation of luncheon meats. Brazilian Journal of Food Technology, 18, 49–56. 10.1590/1981-6723.3814

[fsn31880-bib-0013] Cunha, L. C. M. , Monteiro, M. L. G. , Lorenzo, J. M. , Munekata, P. E. S. , Muchenje, V. , de Carvalho, F. A. L. , & Conte‐Junior, C. A. (2018). Natural antioxidants in processing and storage stability of sheep and goat meat products. Food Research International, 111, 379–390. 10.1016/j.foodres.2018.05.041 30007699

[fsn31880-bib-0014] Delgado, G. T. C. , Tashiro, W. M. S. C. , Maróstica Júnior, M. R. , & Pastore, G. M. (2013). Yacon (*Smallanthus sichifolius*): A functional food. Plant Foods for Human Nutrition, 68, 222–228. 10.1007/s11130-013-0362-0 23709016

[fsn31880-bib-0015] Doménech‐Asensi, G. , García‐Alonso, F. J. , Martínez, E. , Santaella, M. , Martín‐Pozuelo, G. , Bravo, S. , & Periago, M. J. (2013). Effect of the addition of tomato paste on the nutritional and sensory properties of mortadella. Meat Science, 93, 213–219. 10.1016/j.meatsci.2012.08.021 22999311

[fsn31880-bib-0016] Domínguez, R. , Pateiro, M. , Gagaoua, M. , Barba, F. J. , Zhang, W. , & Lorenzo, J. M. (2019). A comprehensive review on lipid oxidation in meat and meat products. Antioxidants, 8, 1–31. 10.3390/antiox8100429 PMC6827023 31557858

[fsn31880-bib-0017] Dutra, M. P. , Palhares, P. C. , Silva, J. R. O. , Ezequiel, I. P. , Ramos, A. L. S. , Perez, J. R. O. , & Ramos, E. M. (2013). Technological and quality characteristics of cooked ham‐type pâté elaborated with sheep meat. Small Ruminant Research, 115, 56–61. 10.1016/j.smallrumres.2013.08.007

[fsn31880-bib-0018] Echegaray, N. , Gómez, B. , Barba, F. J. , Franco, D. , Estévez, M. , Carballo, J. , … Lorenzo, J. M. (2018). Chestnuts and by‐products as source of natural antioxidants in meat and meat products: A review. Trends in Food Science & Technology, 82, 110–121. 10.1016/j.tifs.2018.10.005

[fsn31880-bib-0019] Franco, B. D. G. M. , & Landgraf, M. (2005). Microbiologia dos Alimentos. São Paulo, SP: Editora Atheneu.

[fsn31880-bib-0020] Gomes, J. C. , & Oliveira, G. O. (2011). Análises físicas químicas de alimentos. Viçosa, MG: Editora UFV.

[fsn31880-bib-0021] Gómez, B. , Barba, F. J. , Domínguez, R. , Putnik, P. , Bursać Kovačević, D. , Pateiro, M. , … Lorenzo, J. M. (2018). Microencapsulation of antioxidant compounds through innovative technologies and its specific application in meat processing. Trends in Food Science & Technology, 82, 135–147. 10.1016/j.tifs.2018.10.006

[fsn31880-bib-0023] Guerra, I. C. D. , Félex, S. S. S. , Meireles, B. R. L. A. , Dalmás, P. S. , Moreira, R. T. , Honório, V. G. , … Madruga, M. S. (2011). Evaluation of goat mortadella prepared with different levels of fat and goat meat from discarded animals. Small Ruminant Research, 98, 59–63. 10.1016/j.smallrumres.2011.03.019

[fsn31880-bib-0024] Guerra, I. C. D. , Meireles, B. R. L. A. , Félex, S. S. S. , Conceição, M. L. , Souza, E. L. , Benevides, S. D. , & Madruga, M. S. (2012). Carne de ovinos de descarte na elaboração de mortadelas com diferentes teores de gordura suína. Ciência Rural, 42, 2288–2294. 10.1590/S0103-84782012005000113

[fsn31880-bib-0025] Huang, S. C. , Tsai, Y. F. , & Chen, C. M. (2011). Effects of wheat fiber, oat fiber, and inulin on sensory and physico‐chemical properties of Chinese‐style sausages. Asian Australasian Journal of Animal Sciences, 24, 875–880. 10.5713/ajas.2011.10317

[fsn31880-bib-0026] Leite, A. , Rodrigues, S. , Pereira, E. , Paulos, K. , Oliveira, A. F. , Lorenzo, J. M. , & Teixeira, A. (2015). Physicochemical properties, fatty acid profile and sensory characteristics of sheep and goat meat sausages manufactured with different pork fat levels. Meat Science, 105, 114–120. 10.1016/j.meatsci.2015.03.015 25839884

[fsn31880-bib-0027] Lima Júnior, D. M. , Rangel, A. H. N. , Urbano, S. A. , & Moreno, G. M. B. (2013). Oxidação lipídica e qualidade de carne ovina. Acta Veterinaria Brasilica, 7, 14–28.

[fsn31880-bib-0028] Lorenzo, J. M. , Pateiro, M. , Domínguez, R. , Barba, F. J. , Putnik, P. , Kovačević, D. B. , … Franco, D. (2018). Berries extracts as natural antioxidants in meat products: A review. Food Research International, 106, 1095–1104. 10.1016/j.foodres.2017.12.005 29579903

[fsn31880-bib-0029] Maia Junior, J. A. , Henry, F. C. , Valle, F. R. A. F. , Martins, M. L. L. , Quirino, C. R. , & Costa, R. S. (2013). Reducing fat and sodium content in pork sausage. African Journal of. Biotechnology, 12, 3847–3853. 10.5897/AJB2012.11919

[fsn31880-bib-0030] Méndez‐Zamora, G. , García‐Macías, J. A. , Santellano‐Estrada, E. , Chávez‐Martínez, A. , Durán‐Meléndez, L. A. , Silva‐Vásquez, R. , & Quintero‐Ramos, A. (2015). Fat reduction in the formulation of frankfurter sausages using inulin and pectin. Food Science and Technology, 35, 25–31. 10.1590/1678-457X.6417

[fsn31880-bib-0031] Mendoza, E. , García, M. L. , Casas, C. , & Selgas, M. D. (2001). Inulin as fat substitute in low fat, dry fermented sausages. Meat Science, 57, 387–393. 10.1016/S0309-1740(00)00116-9 22061711

[fsn31880-bib-0032] Ojansivu, I. , Ferreira, C. L. , & Salminen, S. (2011). Yacon, a new source of prebiotec oligosaceharides with a history of safe use. Trends in Food Science and Technology, 22, 40–46. 10.1016/j.tifs.2010.11.005

[fsn31880-bib-0033] Oliveira, R. F. , Henry, F. C. , Valle, F. , Oliveira, D. B. , Santos Junior, A. C. , Resende, E. D. , … Martins, M. L. L. (2020). Effect of the fruit aqueous extract of Brazilian pepper tree (*Schinus terebinthifolius*, Raddi) on selected quality parameters of frozen fresh pork sausage. Journal of Agriculture and Food Research, 2, 100055 10.1016/j.jafr.2020.100055

[fsn31880-bib-0034] Osório, J. C. S. , Osório, M. T. M. , & Sañudo, C. (2009). Características sensoriais da carne ovina. Revista Brasileira De Zootecnia, 38, 292–300. 10.1590/S1516-35982009001300029

[fsn31880-bib-0035] Pardi, M. C. , Santos, I. F. , Souza, E. R. , & Pardi, H. S. (1996). Ciência, higiene e tecnologia da carne (1st ed). Goiânia, GO: CEGRAF/UFG.

[fsn31880-bib-0036] Pateiro, M. , Vargas, F. C. , Chincha, A. A. I. A. , Sant'Ana, A. S. , Strozzi, I. , Rocchetti, G. , … Lorenzo, J. M. (2018). Guarana seed extracts as a useful strategy to extend the shelf life of pork patties: UHPLC‐ESI/QTOF phenolic profile and impact on microbial inactivation, lipid and protein oxidation and antioxidant capacity. Food Research International, 114, 55–63. 10.1016/j.foodres.2018.07.047 30361027

[fsn31880-bib-0037] Prior, R. L. , Hoang, H. , Gu, L. , Wu, X. , Bacchiocca, M. , & Howard, L. (2003). Assays for hydrophilic and lipophilic antioxidant capacity (oxygen radical absorbance capacity (ORACFL) of plasma and other biological and food samples. Journal of Agricultural and Food Chemistry, 51, 3273–3279. 10.1021/jf0262256 12744654

[fsn31880-bib-0038] Radha Krishnan, K. , Babuskin, S. , Azhagu, P. S. B. , Sasikala, M. , Sabina, K. , Archana, G. , … Sukumar, M. (2014). Antimicrobial and antioxidant effects of spice extracts on the shelf life extension of raw chicken meat. International Journal of Food Microbiology, 171, 32–40. 10.1016/j.ijfoodmicro.2013.11.011 24308943

[fsn31880-bib-0040] Sánches‐Rodríguez, M. E. (2001). Parâmetros de color de jamón ibérico de bellota Guijuello al final del período de maturación. Alimentaria, 1, 33–39.

[fsn31880-bib-0041] Santamaria, P. (2006). Nitrate in vegetables: Toxicity, content, intake and EC regulation. Journal of the Science of Food and Agriculture, 86, 10–17. 10.1002/jsfa.2351

[fsn31880-bib-0042] Santana, I. , & Cardoso, M. H. (2008). Raiz tuberosa de yacon (*Smallanthus sonchifolius*): Potencialidade de cultivo, aspectos tecnológicos e nutricionais. Ciência Rural, 30, 898–905. 10.1590/S0103-84782008000300050

[fsn31880-bib-0043] Santos Júnior, A. C. S. , Maia Júnior, J. A. , Henry, F. C. , Oliveira, D. B. , Quirino, C. R. , Martins, M. L. L. , … Cabral, N. O. (2017). Preparation and physico‐chemical characterization of mutton mortadella supplemented with yacón meal. REDVET ‐ Revista Electrónica De Veterinaria, 18, 1–10.

[fsn31880-bib-0044] SAS (2009). User’s guide statistics. Cary: INSTITUTE SAS.

[fsn31880-bib-0045] Silva, N. , Junqueira, V. C. A. , & Silveira, N. F. A. (2007). Manual de métodos de análise microbiológica de alimentos (3rd ed). São Paulo: Livraria Varela.

[fsn31880-bib-0046] Sorensen, G. , & Jorgensen, S. S. A. (1996). Critical examination of some experimental variables in the 2‐thiobarbituric acid (TBA) test for lipid oxidation in meat products. Zeitschrift Für Le0bensmittel‐Untersuchung Und –forschung, 202, 205–210.

[fsn31880-bib-0047] Teixeira, J. T. (2010). Elaboração de apresuntado formulado com farinha e extrato de yacon (Smallanthus sonchifollius). MSc dissertation, Universidade Federal de Lavras, Lavras, MG, Brazil.

[fsn31880-bib-0048] Vidal, A. M. A. , Dias, D. O. , Martins, E. S. M. M. , Oliveira, R. S. , Nascimento, R. M. S. , & Correia, M. G. S. (2012). A ingestão de alimentos funcionais e sua contribuição para a diminuição de incidências de doenças. Caderno De Graduação – Ciências Biológicas E Da Saúde, 1, 43–52.

[fsn31880-bib-0049] Viuda‐Martos, M. , Ruiz‐Navajas, Y. , Fernández‐López, J. , & Pérez‐Álvarez, J. A. (2010). Effect of added citrus fibre and spice essential oils on quality characteristics and shelf‐life of mortadella. Meat Science, 85, 568–576. 10.1016/j.meatsci.2010.03.007 20416839

[fsn31880-bib-0050] Vyncke, W. (1970). Direct determination of the thiobarbituric acid value in trichloracetic acid extracts of fish as a measure of oxidative rancidity. Fette Scifen Anstrichnittel, Leinfelden‐Echterdingen, 72, 1084–1087. 10.1002/lipi.19700721218

[fsn31880-bib-0051] Wu, C. , Duckett, S. K. , Neel, J. P. S. , Fontenot, J. P. , & Clapham, W. M. (2008). Influence of finishing systems on hydrophilic and lipophilic oxygen radical absorbance capacity (ORAC) in beef. Meat Science, 80, 662–667. 10.1016/j.meatsci.2008.03.003 22063579

[fsn31880-bib-0052] Yetim, H. , Gokalp, H. Y. , Kaya, M. , Yanar, M. , & Ockerman, H. W. (1992). Physical, chemical and organoleptic characteristics of Turkish style frankfurters made with an emulsion containing turkish soy flour. Meat Science, 31, 43–56. 10.1016/0309-1740(92)90071-B 22059509

[fsn31880-bib-0053] Zamuz, S. , López‐Pedrouso, M. , Barba, F. J. , Lorenzo, J. M. , Domínguez, H. , & Franco, D. (2018). Application of hull, bur and leaf chestnut extracts on the shelf‐life of beef patties stored under MAP: Evaluation of their impact on physicochemical properties, lipid oxidation, antioxidant, and antimicrobial potential. Food Research International, 112, 263–273. 10.1016/j.foodres.2018.06.053 30131137

[fsn31880-bib-0054] Zeola, N. M. B. L. , Souza, P. A. , Souza, H. B. A. , Silva Sobrinho, A. G. , & Barbosa, J. C. (2007). Cor, capacidade de retenção de água e maciez da carne de cordeiro maturada e injetada com cloreto de cálcio. Arquivo Brasileiro De Medicina Veterinária E Zootecnia, 59, 1058–1066. 10.1590/S0102-09352007000400036

